# NMRK2 Gene Is Upregulated in Dilated Cardiomyopathy and Required for Cardiac Function and NAD Levels during Aging

**DOI:** 10.3390/ijms22073534

**Published:** 2021-03-29

**Authors:** Cynthia Tannous, Robin Deloux, Ahmed Karoui, Nathalie Mougenot, Dean Burkin, Jocelyne Blanc, Dario Coletti, Gareth Lavery, Zhenlin Li, Mathias Mericskay

**Affiliations:** 1 Inserm Unit UMR-S 1180 CARPAT, Faculty of Pharmacy, Université Paris-Saclay, 92296 Châtenay-Malabry, France; cynthiajtannous@gmail.com (C.T.); robin.deloux@gmail.com (R.D.); ahmed.karoui@inserm.fr (A.K.); 2 INSERM Unit U1164 / CNRS UMR 8256, Biologie de l’Adaptation et du Vieillissement, Institut de Biologie Paris-Seine, Sorbonne Université, 75006 Paris, France; Jocelyne.blanc@sorbonne-universite.fr (J.B.); dario.coletti@sorbonne-universite.fr (D.C.); zhenlin.li@sorbonne-universite.fr (Z.L.); 3 Department of Pharmacology and Toxicology, Faculty of Medicine, American University of Beirut Medical Center, Beirut 1107 2020, Lebanon; 4 Plateau d’Expérimentation Cœur, Muscle, Vaisseaux PECMV, UMS28, Sorbonne Université, 75013 Paris, France; Nathalie.mougenot@sorbonne-universite.fr; 5 Department of Pharmacology, Reno School of Medicine, University of Nevada, Reno, NV 89102, USA; dburkin@med.unr.edu; 6 Institute of Metabolism and Systems Research, University of Birmingham, 2nd Floor IBR Tower, Edgbaston, Birmingham B15 2TT, UK; G.G.Lavery@bham.ac.uk

**Keywords:** dilated cardiomyopathy, nicotinamide adenine dinucleotide, nicotinamide riboside kinase 2, muscle integrin binding protein, eccentric hypertrophy, pressure overload

## Abstract

Dilated cardiomyopathy (DCM) is a disease of multifactorial etiologies, the risk of which is increased by male sex and age. There are few therapeutic options for patients with DCM who would benefit from identification of common targetable pathways. We used bioinformatics to identify the *Nmrk2* gene involved in nicotinamide adenine dinucleotde (NAD) coenzyme biosynthesis as activated in different mouse models and in hearts of human patients with DCM while the *Nampt* gene controlling a parallel pathway is repressed. A short NMRK2 protein isoform is also known as muscle integrin binding protein (MIBP) binding the α7β1 integrin complex. We investigated the cardiac phenotype of Nmrk2-KO mice to establish its role in cardiac remodeling and function. Young Nmrk2-KO mice developed an eccentric type of cardiac hypertrophy in response to pressure overload rather than the concentric hypertrophy observed in controls. *Nmrk2-KO* mice developed a progressive DCM-like phenotype with aging, associating eccentric remodeling of the left ventricle and a decline in ejection fraction and showed a reduction in myocardial NAD levels at 24 months. In agreement with involvement of NMRK2 in integrin signaling, we observed a defect in laminin deposition in the basal lamina of cardiomyocytes leading to increased fibrosis at middle age. The *α7 integrin* was repressed at both transcript and protein level at 24 months. *Nmrk2* gene is required to preserve cardiac structure and function, and becomes an important component of the NAD biosynthetic pathways during aging. Molecular characterization of compounds modulating this pathway may have therapeutic potential.

## 1. Introduction

Dilated Cardiomyopathy (DCM) is a severe heart disease characterized by the dilation of the left and right ventricles leading to a systolic dysfunction and reduced ejection fraction, without hypertension, pressure overload or coronary artery disease sufficient to cause global systolic impairment [1]. DCM is the principal cause of heart transplantation in the western world [2]. There are genetic and non-genetic causes of DCM. Mutations in 30 genes encoding mostly but not exclusively cytoskeletal proteins, have been identified in monogenic forms of DCM though modern high-throughput genome sequencing approaches, suggesting that multiple gene variants may be involved in some cases [1,3]. Non-genetic causes include exposure to toxins, cancer drugs or enteroviral infection (myocarditis), however, susceptibility to DCM is predominantly modulated by genetic mechanisms [1].

A major issue for the development of therapeutic molecules for rare diseases like DCM resides in the fact that developing drugs for each different subtype of patients with different genetic etiologies is a risky and complex strategy. An appealing alternative approach is to identify therapeutic targets that will be common to DCM with different etiologies to target the largest number of patients. A common feature to most forms of DCM is concurrent structural eccentric remodeling of the cardiac chambers and underlying changes in cardiac metabolism [4]. The expression of cardiac genes encoding sarcomeric proteins, cytoskeleton linkers and receptors, such as the integrin receptors and dystrophin-glycoprotein complex connected to the extracellular matrix (ECM) [5,6], as well as genes involved in the regulation of energy metabolism are commonly perturbated in DCM [7]. With the aim of identifying pathways common to different forms of DCM, we analyzed and compared publicly available transcriptomic data sets for different DCM models. We identified a gene with two different annotations in different databases, integrin β1 binding protein 3 (*Itgb1bp3*) or nicotinamide riboside kinase 2 (*Nmrk2*, official symbol), as a robustly upregulated in several etiologically distinct mouse models of DCM [8,9].

Initial functional characterizations focused on the protein product of *Itgb1bp3* (also named Muscle Integrin Binding Protein (MIBP)), identified in a yeast two-hybrid screen of a human heart cDNA library for the β1 integrin cytoplasmic tail [10]. MIBP was detected only in striated cardiac and skeletal muscle tissues. Yet, overexpression of MIBP in C2C12 skeletal muscle cell line disrupted the formation of myotubes [10]. MIBP interacts with the α7β1 integrin heterodimer and stimulates the phosphorylation of paxillin, an integrin binding protein, and inhibits the deposition of laminin in the extracellular matrix (ECM) [11]. Mutation of the zebrafish homologue *Nrk2b gene* also causes a defect in the deposition of laminin and abnormal muscle morphology suggesting that the expression of *Nmrk2/Itgb1bp3* is precisely regulated for coherent morphogenesis of muscle and extracellular matrix [12,13].

Similarly, a conserved eukaryotic NAD^+^ biosynthetic pathway mediated by the nicotinamide riboside kinase gene *Nrk1* in yeast and *Nmrk1* and *Nmrk2* genes in humans has also been identified [14]. *Nmrk2* was described as encoding a longer 230 amino acid (aa) splice variant (26 kDa) of the previously described 186 aa MIBP protein. Full length NMRK2 and NMRK1 phosphorylate the vitamin B3 precursor nicotinamide riboside (NR) to generate nicotinamide mononucleotide (NMN), an immediate precursor of NAD^+^ in the biosynthetic pathway. The 186 aa MIBP lacks the catalytic domain. NR phosphorylation by NMR-Kinases represents one the four different biosynthetic pathways for NAD^+^ described in eukaryotes that include two other vitamin B3s, nicotinic acid (Na) and nicotinamide (NAM) and the tryptophan (TRP) amino acid [15].NAD^+^ is a major hydride transfer coenzyme in the oxidation of energy substrates, as well as a co-substrate used by enzymes such as the Sirtuins deacetylases, the poly(ADPribose) polymerases (PARPs), and the CD38 ADPribose cyclase, regulating energy metabolism, resistance to oxidative stress and calcium signaling, respectively [16,17].All these enzymes cleave NAD^+^ into ADP-ribose and nicotinamide (NAM). NAM can be recycled into NMN by the nicotinamide phosphoribosyl transferase (NAMPT), which plays a major role in the maintenance of tissue NAD levels in mammals but is repressed in the failing heart [18,19].

Hence, *Nmrk2* could be involved both in **i)** integrin signaling and extracellular matrix composition, which are major altered mechanisms in DCM; **ii)** synthesis of NAD^+^, whose functions as a coenzyme and a signaling molecule are central to energy metabolism. We have previously explored the skeletal muscle phenotype of NMRK2-deficient mice, showing the involvement of this kinase in salvage of exogenous NR and adaptation to exercise [20,21]. In the present study, we explore the cardiac phenotype of NMRK2-deficient mice showing that *Nmrk2* has a dual role in cardiac structure and metabolic function during aging.

## 2. Results

### 2.1. Nmrk2 Upregulation Is a Common Signature in Mouse Models of DCM and in Humans

**Figure 1:**
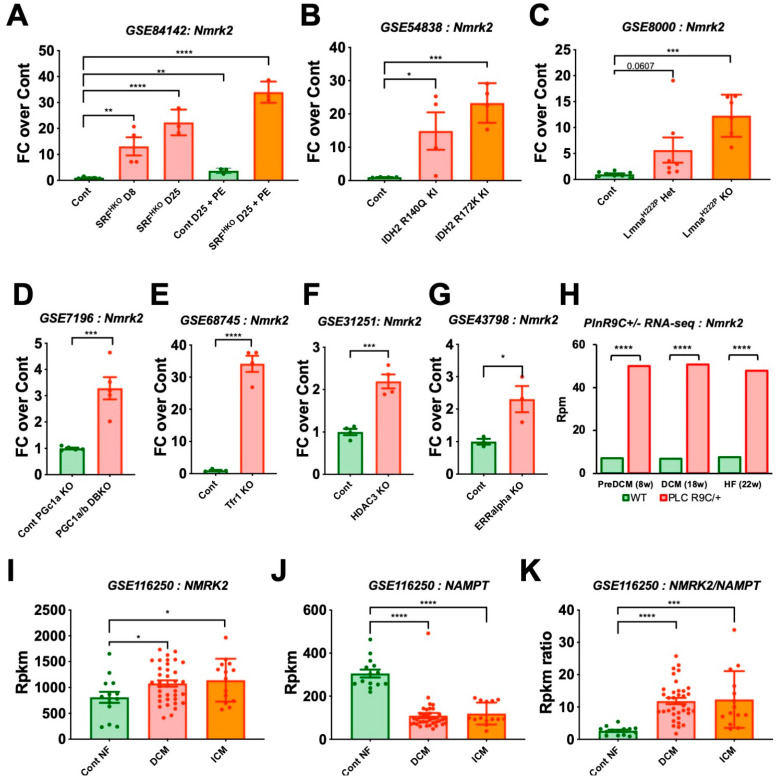
*Nmrk2* is upregulated in different models of dilated Cardiomyopathy (DCM). Public Table 4. month-old adult mice analyzed at day (**A**) 8 and D25 after cardiac-specific deletion of the Serum Response Factor gene (SRF^HKO^ mutant), or D25 with phenylephrine administration (80 mg/kg/day, 15 days), and controls (*n* = 3 to 5 mice/group) [9]. (**B**) Adult mice analyzed at 1 month after conditionally-activated expression of isocitrate dehydrogenase 2 mutants (IDH2) R140Q and R172K (*n* = 4 mice/group) [22]. (**C**) A-type lamin Lmna H222P homo-/heterozygous mutants at 10 weeks of age, an early pre-symptomatic stage (*n* = 6 to 8 mice/group) [23]. (**D**) 2 to 3-month-old adult mice doubly deficient for peroxisome proliferator-activated receptor γ coactivators *Pgc-1*α (global KO) and *Pgc-1β* (cardiac-specific KO, 1 month after deletion) versus *Pgc-1*α KO (*n* = 4 mice per group) [25]. (**E**) 10-days-old pups with cardiac-specific deletion of the transferrin receptor 1 gene (*Tfr1*) (*n* = 4 mice/group) [26]. (**F**) 6-week-old mutants deleted for histone deacetylase 3 (HDAC3). Deletion occurred 7 days after birth (*n* = 4 mice/group) [27]. (**G**) 2 to 3-month-old adult mice lacking estrogen-related receptor alpha (ERRalpha) (*n* = 3 mice/group) [28]. (**H**) Adult mice expressing a missense mutation (p.Arg9Cys) in phospholamban (PLNR9C/+) analyzed at early asymptomatic pre-DCM stage (8 weeks), DCM stage (18 weeks) and HF stage (22 weeks). RNA was extracted from purified cardiomyocytes, pool of 3 mice/group, *p* values are indicated as from supplementary table in reference [7]. (**I–K**) RNA-seq data from human patients, non-failing (NF, *n* = 14) controls, DCM (*n* = 37) and ischemic cardiomyopathy (ICM, *n* = 13) [31]. (**I**) *NMRK2* and (**J**) Nicotinamide phosphoribosyl transferase (*NAMPT)* expression level; (**K**) *NMRK2* on *NAMPT* expression ratio. Dots are values for each individual in A to I. Data are shown as mean fold change over mean control value (colored bars) +/- SEM (error bars) in I to K. GEO dataset reference is indicated in each panel. Statistics: Only comparison of each mutant group against the control groups were planned. t-test: *, *p* ≤ 0.05; **, *p* ≤ 0.01 ***; *p* ≤ 0.001; ****; *p* ≤ 0.0001.

To understand in which type of heart disease the *Nmrk2* gene expression profile was modulated, we searched in the Gene Expression Omnibus (GEO) functional genomics data repository using “Nmrk2” and “heart” as keywords. *Nmrk2* gene was strikingly upregulated in a number of mouse genetic models of DCM with very different etiologies ranging from heart specific serum response factor (SRF) transcription factor deletion (SRF^HKO^) which displays reduced sarcomeric and energy transfer gene expression [9] (Figure 1A) to isocitrate dehydrogenase 2 (IDH2) mutations that alter intermediary metabolism and energy production [22] (Figure 1B), lamin A/C (LMNA) mutation that perturbate nuclear lamina structure and signaling [23,24] (Figure 1C), double deletion of the PPARγ coactivators (PGC) 1α and β that are required for mitochondrial biogenesis [25] (Figure 1D), deletion of the transferrin receptor (TFR1) regulating iron metabolism [26] (Figure 1E), deletion of the histone deacetylase 3 (HDAC3) that sensitizes the heart to high fat diet-induced cardiomyopathy and mitochondrial dysfunction [27] (Figure 1F), and deletion of the estrogen related receptor (ERR) that interacts with PGC1 to activate mitochondrial biogenesis [28] (Figure 1G). RNA-seq analysis of the phospholamban (PLN) R9C model of heart failure (HF) showed that like in the SRF^HKO^ model, upregulation of *Nmrk2* gene is an early event occurring prior the development of overt DCM and HF [7] (Figure 1H). *Nmrk2* was also reported as the top upregulated gene (FC 16.87, *p* < 0.001) in glycogen synthase kinase 3 (GSK-3)–deficient hearts, which is involved in cardiac cells homeostasis and energy metabolism [29,30].

In humans, the baseline expression of *NMRK2* gene, 800 reads per kilobase per million (Rpkm) in non-failing heart (Figure 1I) is higher than in mice (Figure 1H, see wildtype (WT) controls), and the increase in expression is more modest in human DCM patients [31]. However, it is contrasted by a clear decrease in the expression of the gene encoding the nicotinamide phosphoribosyl transferase (*NAMPT*) (Figure 1J), as reported previously [9]. Consequently, there is a shift toward higher *NMRK2/NAMPT* ratio in the failing heart from DCM patients compared to controls (Figure 1K). Altogether these observations show that the expression of the *Nmrk2* gene is increased in response to perturbations in cardiac energy and contractility and raises the question of its role in the heart.

### 2.2. Nmrk2 Is a Striated Muscle Specific Protein Associated to the Sarcolemna in Control Hearts and Delocalized to the Cytosol When Overexpressed in the Failing Heart of SRF^HKO^ Mice

**Figure 2:**
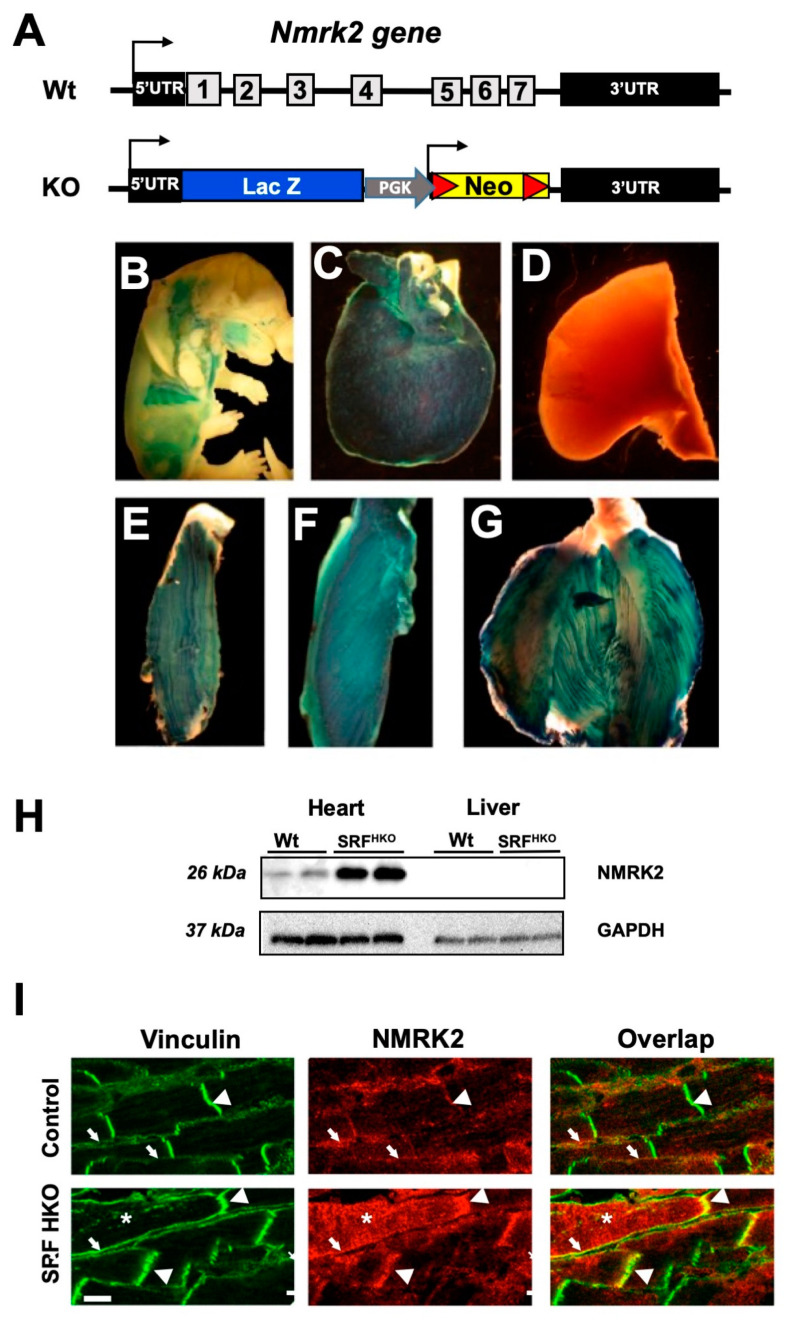
Nmrk2 gene knock-out design and expression pattern. (**A**) Definitive null allele was obtained by replacement of all exons and introns of the *Nmrk2* gene by an expression cassette including the lacZ reporter gene and a floxed neomycin resistance gene. (**B**–**G**) The *lacZ* gene, which is under the control of *Nmrk2* regulatory sequences *β*-galactosidase staining at embryonic day 14.5 (**B**), in the adult heart (**C**)**,** the liver (negative) (**D**) and the skeletal muscles; plantaris (**E**), soleus (**F**) and gastrocnemius (**G**) (representative picture of *n* = 3 mice/group). (**H**) Western blot for NMRK2 protein in indicated tissues from control and *SRF-HKO* mice. (**I**) Confocal microscopy of cardiac sections stained with anti-h1-Vinculin antibody (green) and anti-NMRK2 antibody (red) in control mice and dilated heart from *SRF-HKO* mice (representative picture of *n* = 3 mice/group). Bottom left white Bar = 10 µm, arrowheads: intercalated disks (stretched in *SRF-HKO)*, arrows: lateral sarcolemma, asterisks: cytosol filled with NMRK2.

To analyze the function of the *Nmrk2* gene in vivo, all the 7 exons of the gene were replaced by a cassette including a *LacZ* reporter gene and a neomycin resistance gene via homologous recombination in embryonic stem (ES) cells (Figure 2A). Heterozygous *Nmrk2* +/- mice were obtained among chimeric founder offspring and bred together. Homozygous *Nmrk2* -/- (KO) male and female mice were born at the expected 25% Mendelian ratio and were viable and fertile. Since the *LacZ* reporter gene is placed immediately downstream of the 5′-untranslated region of the *Nmrk2* gene, its expression pattern reflects the activity of the *Nmrk2* promoter. *LacZ* expression was not detected at the early stages of embryonic development from 9.5 to 12.5 days post-coïtum (d.p.c.) and started in developing muscle masses only at the fetal stage (Figure 2B). *LacZ* expression was detected exclusively in the heart and skeletal muscles in 2 months-old *Nmrk2* +/- mice, (Figure 2C–G). No activity was detected in brain, lungs and liver (Figure 2D* and data not shown*). Low level of NMRK2 protein was detected by western at baseline in the adult heart and the signal was robustly increased in the failing heart of the *SRF^HKO^* mice (Figure 2H). NMRK2 was not detected in the liver. To determine its subcellular localization in the myocardium, immunofluorescent staining was performed for NMRK2 and for vinculin, a cytoskeletal linker binding to integrins at the costameres, and to cadherins at intercalated disks. A partial overlap of NMRK2 and vinculin was observed in these regions in control hearts (Figure 2I, top panels). NMRK2 was highly increased in the *SRF^HKO^* failing heart (Figure 2I, bottom panels), in which cardiomyocytes are elongated and intercalated disks are stretched and sinuous as previously reported [8,32]. The NMRK2 staining still overlapped with vinculin in this condition although it also extended to the cytosol in a number of cardiomyocytes (Figure 2I, asterisk). Altogether, these observations suggest that NMRK2 is a kinase mostly associated to the sarcolemma at baseline that is overexpressed and delocalized to the cytosol when eccentric cardiac remodeling takes place in the context of DCM.

### 2.3. Eccentric Cardiac Remodeling in Young Adult Mice Lacking Nmrk2 upon Pressure Overload

**Figure 3:**
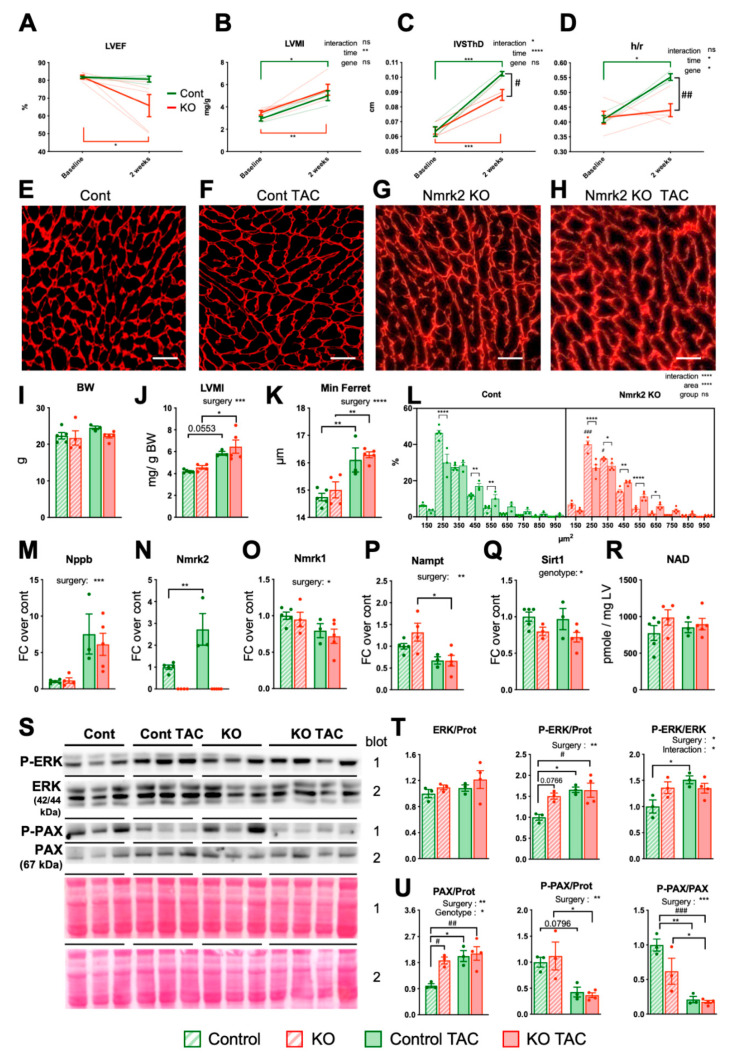
Nmrk2-KO mice develop cardiac hypertrophy in response to transverse aortic constriction. Control mice (*n* = 3) and *Nmrk2-KO* mice (*n* = 5) were subjected to transverse aortic constriction (TAC) and sacrificed 15 days later for histological and molecular analyses. (**A–D**) Echocardiography before surgery (baseline) and after TAC (see accompanying Table 1). (**A**)Left ventricular (LV) ejection fraction. (**B**) LV mass index. **(C)** End-diastolic interventricular septum thickness. (**D**) LV wall thickness to radius ratio (end diastole). Data are shown a mean (full line) +/ SEM. Individual data are shown as dotted lines. (**E**–**H**) Cardiac sections stained with anti-laminin α2 antibody to demarcate cell boundaries, scale bars = 20 µM. (representative picture of *n* = 3 mice per group) (**I**–**R**) Post-sacrifice analyses of controls TAC (*n* = 3) and *Nmrk2-KO TAC* mice (*n* = 5) and age-matched 6 week-old controls (*n* = 4–5) and KO (*n* = 4–3) Body weight. (**J**) Left ventricular mass index at sacrifice. (**K**) Cardiomyocytes diameter (minimum Feret). (**L**) Cardiomyocytes area frequency plot. (**M**–**Q**) Relative expression by RT qPCR of indicated genes. Data normalized on *Gapdh* reference mRNA level. (**R**) Myocardial NAD levels. (**S**) Western blot analysis of ERK and Paxillin (PAX) total and phosphorylated form (P). (**T,U**) Quantification of western blots in panel S using Ponceau staining in the same region of the blot for normalization of loading. Throughout the figure, individual data are plotted as dots (*n* = 3 mice/group), bars and error bars represent mean value and SEM, respectively. In N to Q, T and U, data are expressed as fold changer over mean control value. Two-way repeated measure ANOVA for independent samples was used for A-D panels and two-way ANOVA for I-M and T-U panels: *, *p* ≤ 0.05; **, *p* ≤ 0.01; ***, *p* ≤ 0.001; ****, *p* ≤ 0.0001, as indicated next to graph title. Multiple comparison tests: Šidak for A–D panels, Tukey for I-R and T–U panels: *, *p* ≤ 0.05; **, *p* ≤ 0.01 ***, *p* ≤ 0.001 TAC vs baseline within the same genotype; #, *p* ≤ 0.05, ##; *p* ≤ 0.01; ###, *p* ≤ 0.001, *Nmrk2-KO* mice vs wild type as indicated or between control and *Nmrk2-KO* groups for panel L. t-test for N panel: **, *p* ≤ 0.01 between control and *Nmrk2-KO* groups.

To assess cardiac functions in the *Nmrk2-KO* mice, we performed echocardiography in adult mice at 2 months of age. We observed no differences in left ventricle ejection fraction (LVEF) and cardiac chamber dimensions in systole or diastole (Figure 3A–D and Table 1) at this age. As mentioned, NMRK2 was also known under the name of MIBP (or Itgb1p3) as a protein binding integrin β1. Integrinβ1 partner proteins such as melusin (Itgb1bp2) play an important role in the transduction of biomechanical stress in the heart, especially in the context of transverse aortic constriction (TAC) leading to pressure-overload hypertrophy [33]. To assess the role of NMRK2 kinase in this context, we performed TAC in *Nmrk2-KO* mice and compared the response with control mice subjected to the same procedure. Left ventricle ejection fraction (LVEF) was reduced in *Nmrk2-KO* mice at 2 weeks post-TAC when it was maintained in controls (Figure 3A). The increase in left ventricle mass index (LVMI) estimated by echocardiography was comparable between *Nmrk2-KO* and control mice (Figure 3B) as validated at sacrifice for comparable body weights at this age (Figure 3I,J). The increase in interventricular septum thickness (IVSTh) was less pronounced in *Nmrk2-KO* mice (Figure 3C). LV posterior wall thickness was increased similarly in both genotypes (Table 1). Control mice developed the concentric type of cardiac remodeling as highlighted by the increase in LV thickness-to-radius ratio (h/r) (Figure 3D). In the *Nmrk2-KO* mice, the LV chamber trended to dilate at the same time that myocardium hypertrophy took place and the h/r did not increase (Figure 3D and Table 1). At sacrifice, hearts were harvested, weighted and cardiac sections were processed for immunostaining with anti-laminin α2 antibody delineating the contours of cardiac fibers in order to perform histomorphometric analyses (Figure 3E–H). The laminin α2 staining pattern highlighted a thickening and disorganization of the cardiomyocytes basal lamina in the *Nmrk2-KO* hearts upon TAC (Figure 3H). Cardiomyocyte mean diameter was similarly increased by TAC in both genotypes and the distribution of cardiomyocytes cross-sectional area was not different (Figure 3K,L). The cardiac stress markers *Nppb* was similarly increased in both genotypes (Figure 3M) as were *Nppa* and *skeletal actin* genes (Supplementary Figure S1A,B) and the *Serca2a* gene encoding the sarcoplasmic reticulum calcium pump ATPase was repressed to the same extent (Supplementary Figure S1C). Integrin gene *Itga7* was significantly reduced upon TAC in Nmrk2-KO mice while *Itgb1* expression remained stable in both genotypes (Supplementary Figure S1D,E). TAC increased the level of *Nmrk2* mRNA in control mice (Figure 3N) but decreased the expression of *Nmrk1* and *Nampt* in both control and *Nmrk2-KO* mice (Figure 3O,P). The NAD^+^-dependent deacetylase *Sirt1* was slightly repressed at baseline in the *Nmrk2-KO* mice and remained low upon TAC (Figure 3Q). Myocardial NAD pools (NAD^+^ and NADH) were not altered in *Nmrk2-KO* mice at this age, neither at baseline nor after TAC (Figure 3R). ERK2 MAP kinase was hyperphosphorylated upon TAC in control animals but was already activated at baseline in *Nmrk2-KO* mice and phosphorylation did not increase further upon TAC in this group (Figure 3S,T). Paxillin, which binds and transduces the signal of integrin receptors was hypo-phosphorylated upon TAC in both control and Nmrk2-KO mice (Figure 3S,U). Altogether these results highlight a trend of *Nmrk2-KO* mice to develop a more deleterious type of eccentric cardiac remodeling in response to pressure overload associated with basal lamina defects and reduced LVEF although it does not result in detectable myocardial NAD pool reduction at this stage.

### 2.4. Nmrk2 Mutants Develop Eccentric Cardiac Remodeling and Subclinical LV Dysfunction with Aging

**Figure 4:**
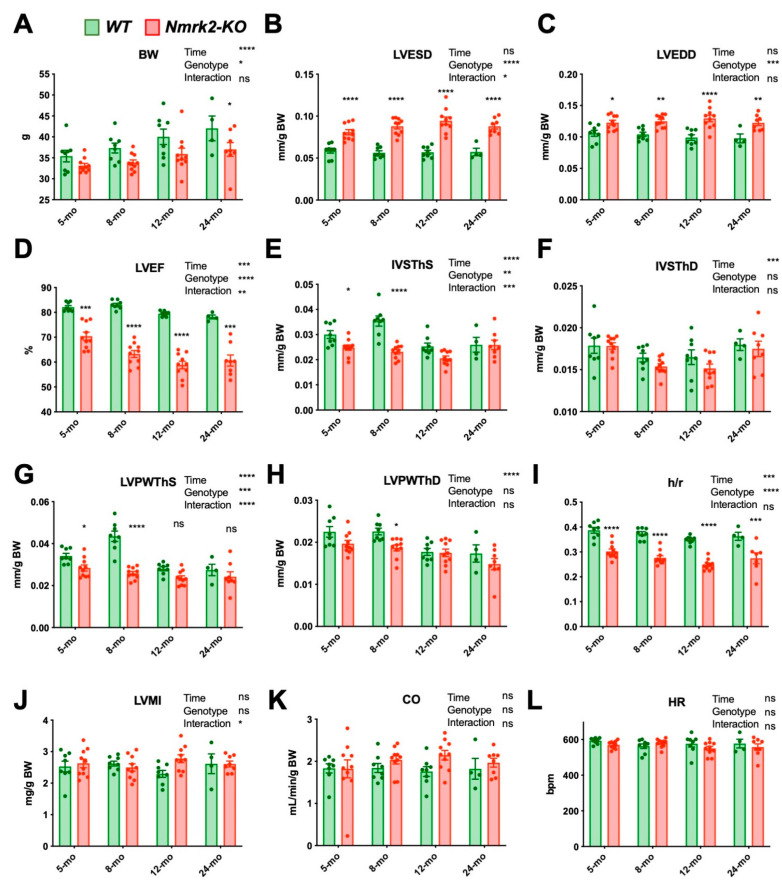
Cardiac dysfunction and left ventricle dilatation take place with aging in the *Nmrk2-KO* mice. Follow-up echocardiography analysis of Wt (*n* = 8) and *Nmrk2-KO* mice (*n* = 10) at 5, 8, 12 and 24 months (mo). (**A**) Body weight. (**B**) LV end-systolic and (**C**) end-diastolic diameter. (**D**) LV ejection fraction. (**E**) End-systolic and (**F**) end-diastolic interventricular septum thickness. (**G**) End-systolic and (**H**) end-diastolic LV posterior wall thickness. (**I**) LV wall thickness to radius ratio (end diastole). (**J**) LV mass index. (**K**) Cardiac output. (**L**) HR, heart rate in beats per minute. Bars and error bars represent the mean value ± SEM Statistics: Mixed model analysis by restricted maximum likelihood (REML). Fixed factors *p* value are indicated next to the graph title: *, *p* ≤ 0.05; **, *p* ≤ 0.01; ***, *p* ≤ 0.001; ****, *p* ≤ 0.0001 for, time, genotype, time x genotype interaction. Sidak’s multiple comparisons test *p*-value: *, *p* ≤ 0.05; **, *p* ≤ 0.01; ***, *p* ≤ 0.001; ****, *p* ≤ 0.0001, between control and *Nmrk2-KO* mice within each age group.

The previous analyses suggested that young *Nmrk2-KO* mice (2 to 3 months-old) have a subclinical phenotype that may evolve with aging. To test this hypothesis, we assessed cardiac function in 12 *Nmrk2-KO* mice and 8 wild-type control mice by serial echocardiography at 5, 8, 12 and 24 months. In the mutant group, two death events occurred before eight months and two animals out of the remaining 10 mice died between 12 and 24 months. In the control group, four out of the eight mice died between 12 and 24 months. Body weight was lower in *Nmrk2-KO* mice during all the survey period (Figure 4A) so cardiac dimensions were adjusted to body weight measured at each age but normalization on tibia length measured after death or sacrifice gave similar results (Supplementary Figure S2). From 5 to 12 months, the LV end-systolic diameter normalized to body weight, progressively increased with aging in the *Nmrk2-KO* mice while it remained stable in controls (Figure 4B) and the normalized LV end-diastolic diameter was larger in *Nmrk2-KO* group independently of the age (Figure 4C Thus, the LVEF declined faster with aging in the *Nmrk2-KO* than in controls (Figure 4D). LV walls passed by phase of growth in control animals from 5 to 8 months before declining at later ages while it remained stable throughout the survey period in *Nmrk2-KO* mice (Figure 4E,G). End-diastolic IVSTh and LPWth evolved with time in a similar pattern in both groups (Figure 4F,H). However, the h/r ratio, which is calculated in end-diastole and independent of body weight, declined with aging in *Nmrk2-KO* mice compared to controls (Figure 4I). LVMI, cardiac output and heart rates were not different between the two groups (Figure 3J–L).

To summarize, adult *Nmrk2-KO* mice develop a subclinical cardiac dysfunction associated with eccentric cardiac remodeling during aging although not evolving towards terminal heart failure in the absence of stress under standard animal house conditions. Morphometric analyses of isolated cardiomyocytes at 4 months of age confirmed those observations eccentric remodeling of the ventricles as Nmrk2-KO myocytes were longer and with higher length-to-width ratio than wildtype myocytes (Supplementary Figure S3).

### 2.5. Nmrk2 Is Required to Preserve NAD Levels in the Heart at Late Age

**Figure 5:**
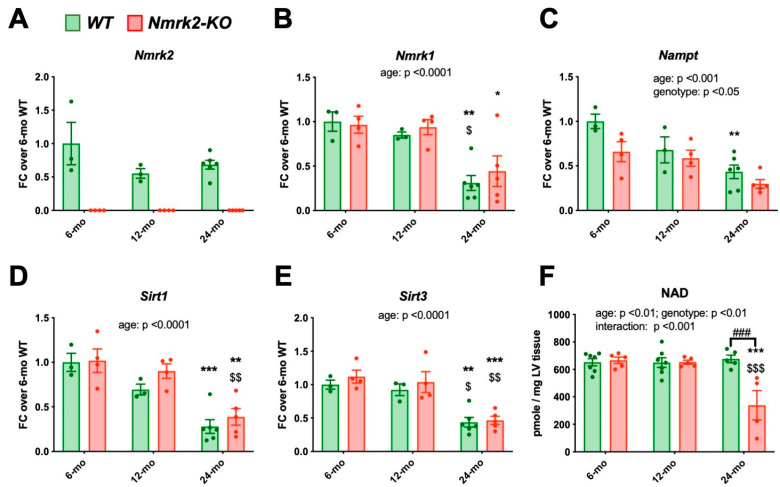
Expression level of genes involved in NAD salvage pathway and quantification of NAD contents in the heart of control and *Nmrk2 KO* mice. (**A–E**) RT-qPCR analysis of cardiac mRNA from control (*n* = 3–6) and *Nmrk2-KO* mice (*n* = 4–5) at the age of 6, 12 and 24 months. Data are normalized on *Gapdh* reference mRNA level and expressed as fold change over the mean value of controls at 6 months. (**F**) Myocardial NAD levels. Individual data are shown by dots. Bars and error bars represent the mean value ± SEM. Statistics: Two-way ANOVA for independent samples: Factors *p* values are indicated are indicated next to the graph title. On bars, Post-hoc Tukey’s multiple comparison test: *, *p* ≤ 0.05; **, *p* ≤ 0.01; ***, *p* ≤ 0.001 24 months vs 6 months within the same genotype; $, *p* ≤ 0.05, $$; *p* ≤ 0.01; $$$; *p* ≤ 0.001, 24 months vs 12 months within the same genotype. ###, *p* ≤ 0.001, *Nmrk2-KO* mice vs wild type within the same age group.

We assessed the consequence of NMRK2 loss on the expression of cardiac genes associated with cardiac aging and disease in independent series of mice at 6, 12 months and 24 months after the last echocardiography. Cardiac *Nmrk2* expression did not decline significantly in control mice (Figure 5A), while both *Nmrk1,* and particularly *Nampt* expression, showed a clear decrease during aging (Figure 5B,C). The levels of *Sirt1* and *Sirt3* decreased similarly in both groups with age (Figure 5D,E). Myocardial NAD pools remained stable overtime in the control hearts whereas they declined between 12 to 24 months in the heart of *Nmrk2-KO* mice (Figure 5F). *Myh7* gene encoding the cardiac, slow *β* isoform of myosin heavy chain, increased progressively in both controls and mutants during aging, but was higher in the mutants at all ages (Supplementary Figure S4A). The age associated increase in *Nppa* expression was similar in both genotypes while *Nppb* remained stable (Supplementary Figure S4B,C). The expression level of *Serca2a* and *Sod1* encoding the superoxide dismutase decreased similarly in both groups with age, as the insulin-regulated glucose transporter *Glut4* (Supplementary Figure S4D–F). *Glut1* increased similarly in both groups (Supplementary Figure S4G). *Pgc1α* and *Acta1* did not change significantly (Supplementary Figure S4H,I).

### 2.6. Laminin Disorganization and Cardiac Fibrosis in Nmrk2-KO Mice

**Figure 6:**
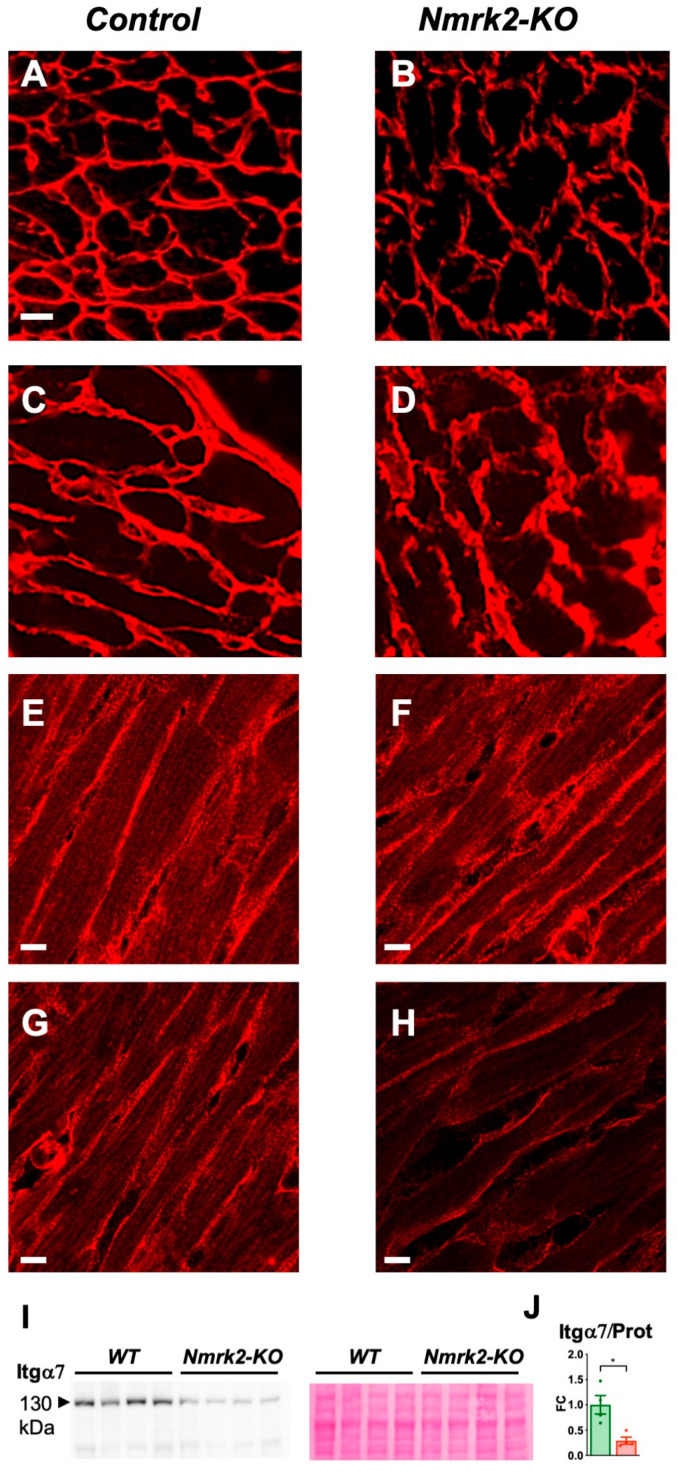
Laminin and integrin α7 pattern in *Nmrk2-KO* myocardium. (**A**–**D**) Laminin α2 staining in frozen heart sections from control and *Nmrk2-KO* mice at the age of 4 months (**A**,**B**) and 24 months (**C**,**D**). (**E**–**H**) Integrin α7 staining from control and *Nmrk2-KO* mice at the age of 4 months (**E**,**F**) and 24 months (**G**,**H**). White bars = 10 µM. Pictures are representative of *n* = 3 mice/group. (**I**) Western blot showing integrin α7 expression in the heart of control and *Nmrk2-KO* mice at 24 months. Red Ponceau staining on the right panel was used for loading control. (**J**) Quantification of western blot. Individual data are plotted (*n* = 4/group). Bar graph represents de fold change over mean control value and error bars the SEM. t-test: *, *p* < 0.05.

**Figure 7:**
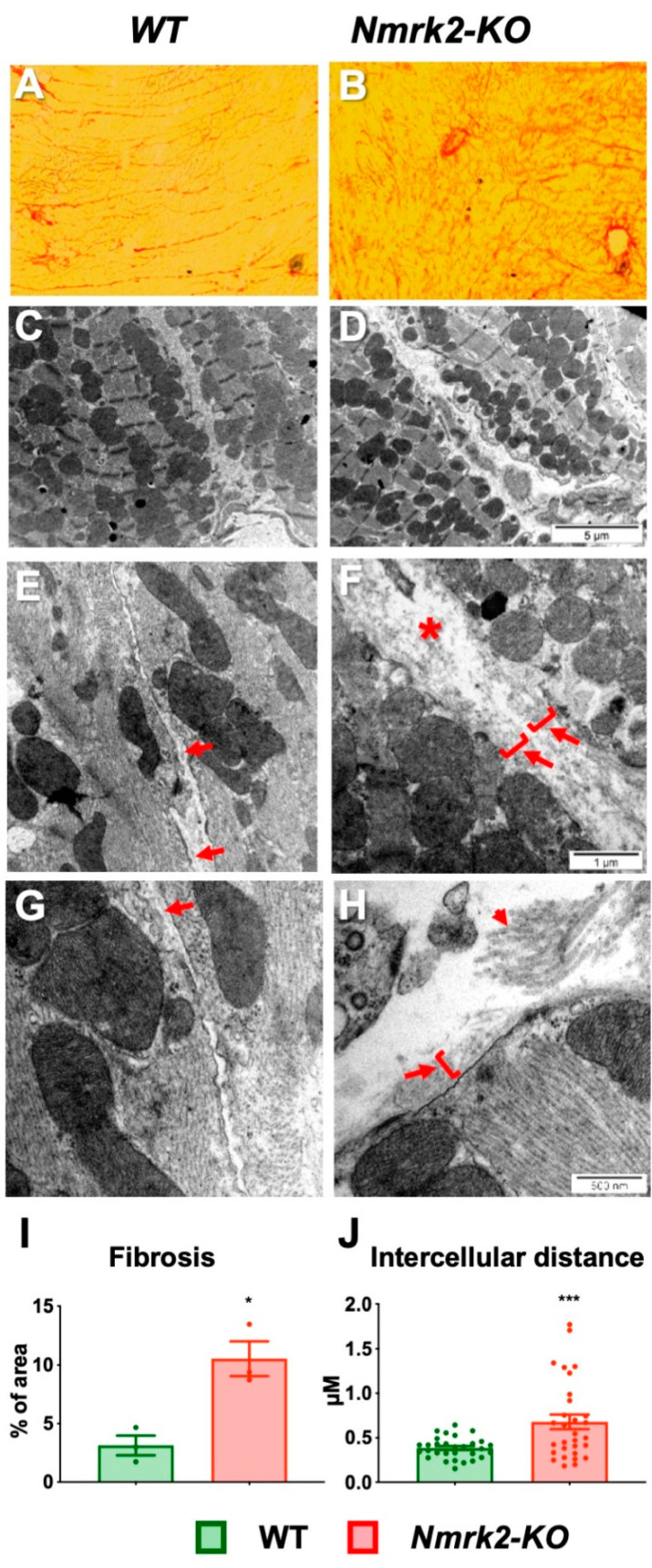
Histological analysis and ultrastructural analysis of cardiac muscle. Heart sections from 12 month-old controls (**A**) and mutant (**B**) were stained with Sirius red. Note the increase of collagen fibers in the region of the atrioventricular junction of the left ventricle in the mutant. Bar = 100 µm. (**E**–**H**) Electron microscopy of heart sections from 12 months-old controls (**C**,**E**,**G**) and mutants (**D**,**F**,**H**) at different magnifications. Asterisks in F highlights the wide extracellular space separating cardiomyocytes in Nmrk2-KO heart sections. Arrows point to the thin basal lamina that run along the plasma membrane in controls (**E**,**G**) while the basal lamina is poorly defined in the *Nmrk2-KO* (**F**,**H**) and intermixed with amorphous extracellular matrix spreading away from the sarcolemma (arrows and brackets). Small arrow in H points to large collagen bundles observed in *Nmrk2-KO* sections but not in controls. From A to H, pictures are representative of *n* = 3 mice/group **(I)** Quantification of red Sirius staining expressed as fractional area of the tissue section. Mean value for each individual are plotted (*n* = 3 for each group). Bar graph represents de fold change over mean control value +/- SEM. (**J**) Quantification of the mean distance between cardiomyocytes. Technical replicates (*n* = 30) were obtained from *n* = 3 mice/group. Data are expressed as the mean minimum diameter of fitting ellipses between cardiomyocytes +/- SEM. (**I**,**J**) t-test. *, *p* < 0.05; ***, *p* ≤ 0.001.

Since the NMRK2 kinase is also known under the name of MIBP (Itgb1bp3), which binds to the heterodimer integrin α7/β1, a receptor for the laminin present in muscle cells basal lamina, we assessed the expression of these genes and the localization of the corresponding proteins in the myocardium. The expression level of the mRNAs encoding integrin α7, integrin β1 and melusin (Itgb1bp2), which like MIBP binds to the cytosolic part of integrin β1, as well as laminin α2, decreased with age but there were no differences between controls and Nmrk2-KO mice (Supplementary Figure S5A–D). Change in NMRK2 (MIBP) levels were reported to alter laminin deposition in C2C12 myotubes and zebrafish [11,12]. We examined the organization of laminin on frozen heart sections in mutant and control mice. The anti-laminin α2 antibody signal delineated a thin and sharply defined basal lamina surrounding the cardiomyocytes and capillaries in the control hearts at 4 months (Figure 6A). In the *Nmrk2-KO* mice at same age, the laminin-stained basal lamina showed a discontinuous pattern (Figure 6B). In aged *Nmrk2-KO* hearts the laminin staining was diffuse and spread over a wider intercellular space compared to controls (Figure 6C,D). Integrin α7 expression highlighted the costameres on the longitudinal side of the cardiomyocytes and the pattern appeared identical in controls and *Nmrk2-KO* hearts at 4 months (Figure 6E,F). Integrinα7 staining was less intense in *Nmrk2-KO* hearts at 24 months compared to controls (Figure 6G,H). We confirmed by western blot analysis the down-regulation of Integrin α7 protein level in the mutant at this late age (Figure 6I,J). By comparison, integrin β1 expression pattern was less affected at 24 months in the *Nmrk2-KO* hearts (Supplementary Figure S6). Red Sirius staining of collagen fibers evidenced the development of cardiac fibrosis in the *Nmrk2-KO* hearts (Figure 7A,B), which was confirmed by quantification (Figure 7I). Electron microscopy analysis showed that the extracellular space separating cardiomyocytes in the *Nmrk2-KO* hearts was enlarged compared to control heart section (Figure 7C–F) and this was confirmed by quantification (Figure 7J). We observed the presence of large collagen fibers in the extracellular space in *Nmrk2-KO* hearts that were not observed to such extent in controls (Figure 7G,H). 

## 3. Discussion

This study aimed to understand the role of *Nmrk2* (also known as *Itgb1bp3* encoding MIBP protein), a striated muscle-specific gene with a potential dual role in NAD^+^ biosynthesis and integrin signaling that is strongly upregulated in the heart in various mouse models of DCM. Here, we showed that the *Nmrk2* gene is a striated muscle-specific gene whose expression starts in late fetal stages. *Nmrk2-KO* mice are viable but develop a progressive cardiac dysfunction and eccentric cardiac remodeling associated with an alteration in laminin deposition in the extracellular matrix starting at 4 months, or that can be triggered by pressure overload in 2 months-old mutants. This perturbation is followed by cardiac fibrosis and structural defect in the adult myocardium. NMRK2 deficiency leads to defects in NAD homeostasis in 24 month aged mice. Consistent with our findings, a recent study reported no obvious cardiac phenotype in young *Nmrk2* KO mice but exaggerated LV chamber dilatation in response to myocardial infarction [34].

### 3.1. Role of Nmrk2 in NAD Biosynthesis

NMRK kinases phosphorylate NR to generate NMN, an immediate precursor of NAD. We found that a lack of NMRK2 does not affect the steady-state level of NAD in the myocardium in young adults, suggesting that NAMPT, as a major contributor to NAD salvage, compensates. Similarly, there was no reduction in tissue NAD levels in the mice knocked out for the ubiquitously expressed gene *Nmrk1*, as measured in the liver, brown adipose tissue (BAT), kidney and skeletal muscles [35] nor was there any reduction in NAD levels in the skeletal muscles of Nmrk1/Nmrk2 double Ko mice [20]. Hence it is clear that NMRK enzymes are not essential for NAD maintenance in young healthy adults. However, we found, that the expression of *Nmrk1* and especially *Nampt* strongly decline with aging of the heart while the expression of Nmrk2 remains stable. Our observations revealed that NMRK2 is required to support the maintenance of NAD levels in the heart at 24 months. At this late age, the major biosynthetic pathways are repressed and in absence of NMRK2 myocardial NAD levels fall by 50%. 

### 3.2. Role of NMRK2 in Laminin Deposition and Integrin Stabilization 

Overexpression of MIBP in skeletal myoblasts [10,11] and knock-out of the zebrafish *Nrk2b* gene [12] have both been shown to impact on laminin deposition in the basal membrane of muscle cells. We show an aberrant pattern of laminin organization in the extracellular matrix of the myocardium as one of the earliest defects in *Nmrk2-KO* mice, suggesting a conserved function for this gene throughout evolution in signaling for laminin deposition although this seems to occur only in cardiac muscle as we did not observe such a defect in the skeletal muscles of this mutant in a previous study [21]. The expression of laminin α2, which is the predominant isoform in the basal membrane of cardiomyocytes, is developmentally regulated in both human and rodents [36]. Its levels increases rapidly after birth in line with cardiomyocyte growth before establishing constant lower level expression during adulthood [36]. During the development of cardiac hypertrophy and failure, the imbalance between the increased size of myocyte and the steady state or decreased level of laminin-α2 chain might contribute to the altered sarcolemma properties in the failing heart [36]. A thickening of the laminin layer has been described in the rat heart in isoproterenol-induced cardiac failure, and during hypertrophy induced by myocardial infarction [37]. We observed a similar defect in the laminin organization in the young *Nmrk2-KO* after TAC and during postnatal maturation of the heart between 3 to 4 months when their heart start to dilate and systolic function decreases. Interestingly, mutations in the laminin-α2 gene, which are essentially associated with a phenotype of muscular dystrophy [38] can also be associated with DCM phenotype in some patients [39,40].

The nature of the molecular mechanism linking NMRK2 activity to laminin deposition remains elusive. In the case of the closely related MIBP protein, the selective impact of MIBP overexpression on laminin deposition was linked to its ability to bind to α7β1 integrin, a known receptor for laminin [11]. Interestingly muscular dystrophies similar to those observed in the case of laminin-α2 mutations also arise from mutations affecting the α7β1 integrin heterodimer [41]. This type of impact of an intracellular integrin binding protein on the organization of an extracellular protein may relate to the inside-out integrin activation signals, which, through conformational changes of the integrin transmembrane domains, regulates their affinity for extracellular ligands [42].

The link between NMRK2 kinase activity, converting NR to NMN, and integrin/laminin interactions is not clear. However, supplementing NAD^+^ or its precursors reduces muscle degeneration in zebrafish mutants for integrin α6, α7 or dystroglycan receptors through a paxillin-dependent mechanism impacting on laminin deposition [12,13]. Whether this beneficial effect of NAD^+^ in zebrafish mutants is linked to restoration of the NAD^+^ metabolome and downstream signaling to rescue adhesion of muscle cells, or to a more specific NAD^+^-dependent posttranslational modification of membrane receptors remains unknown. Interestingly, the NAD^+^-dependent ADP-ribosylation of integrin α7 by the ART1 enzyme has been shown to increase its affinity to laminin [43]. The short NMRK2 isoform known as MIBP is devoid of kinase activity [14]. This suggests that when overexpressed as done in a previous study [13], its mode of action could be as a competitor against the binding of the full length NMRK2 to the α7/b1 integrin heterodimer. MIBP overexpression led to a decrease in integrin α7 protein level and an overexpression and hyperphosphorylation of paxillin a mediator of α7/b1 integrin. In *Nmrk2-KO* mice, we show that paxillin levels are increased at baseline in young mice but not its phosphorylation level, and α7 integrin protein is decreased at a late stage in adult heart.

## 4. Materials and Methods

### 4.1. Study Approval

All experiments with animals conformed to the Directive 2010/63/EU of the European Parliament and were approved by the ethics committee Charles Darvin #5 (agreement 00369.01, 5^th^ September, 2015). ES cells heterozygous for the Nmrk2^tm1(KOMP)Vlcg^ allele were obtained from the Knockout Mouse Project Repository (KOMP). Definitive null allele was obtained by replacement of all exons and introns of the *Nmrk2* gene by a lacZ reporter gene followed by a floxed neomycin resistance gene in VGB6 ES cells in C57BL/6NTac genetic background. ES were implanted in C57BL/6N blastocysts at the Centre National de la Recherche Scientifique (CNRS) facility “Typage et archivage animaux modèles” (TAAM), Villejuif, France. Mice were maintained in this background. Homozygous *Nmrk2-KO* mice were viable and fertile. *SRF^HKO^* mice bear the tamoxifen-inducible *α-MHC-MerCreMer* transgene and the *Srf* floxed allele (*Sf/Sf*) as described previously [8]. Tamoxifen (Sigma T5648) was administrated by i.p. injection at a dose of 0.7 mg diluted in 100 µl peanut oil at D0, D1 and D2 to *SRF^HKO^* mice and *Sf/Sf* littermates used as controls.

### 4.2. Transverse Aortic Constriction (TAC)

Anesthesia was induced with 5% isoflurane in 100% oxygen with a delivery rate of 5 l/min until loss of righting reflex. After induction, anesthesia then was maintained with isoflurane 2% in 100% oxygen by intubation with a canula connected to a respirator at 170 strokes (200 μL)/minute. After thoracotomy, TAC was created using 7.0 suture banded between the carotid arteries over a 27-gauge needle. The needle was then gently removed, creating a constriction. Following chest closing and skin suturing, mice were allowed to recover in warm cages and transferred to cages with ad libitum water and food and environmental enrichment. Analgesia consisted of ibuprofen administration in water (0.0275 mg/mL) ad libitum for 24h before and 72 h after surgery. Mice were monitored daily for pain behavior using a mixed score table appearance of fur, weight loss, and behavior criteria. Mice reaching a maximal score of 3 in one of the criteria or a sum of 6 were euthanatized for ethical reasons by intraperitoneal injection of pentobarbital at a dose of 200 mg/kg of body weight.

### 4.3. Statistics

We performed statistical analyses using the Prism 8 software (https://www.graphpad.com, accessed on 10th January 2021). We used unpaired two-tailed Student’s t-test for comparison of two groups or two-way analysis of variance (ANOVA) for comparison of two groups (controls and Nmrk2-KO) with different treatments, followed by Tukey’s post hoc test. For repeated measurements (e.g., echocardiography follow-up) we used two-way repeated measure ANOVA for independent samples followed by Šidak’s multiple comparison tests for groups comparisons. When values were missing, notably in the aging follow-up study, we did not observe a link between the variable observed (e.g., left ventricle ejection fraction) and the loss of individual mice. In that case, we used a mixed model analysis by restricted maximum likelihood (REML) followed by Šidak’s multiple comparison tests. Statistical significance was considered to be *p* < 0.05. 

### 4.4. Supplemental Material and Method

Western blots, qPCR, NAD quantification, histology, confocal immunofluorescence, electron microscopy.

## 5. Conclusions

In conclusion, the link between NMRK2, α7β1 integrin and laminin is now clearly established in different models but further elucidation of precise underlying molecular mechanisms is required. The identification of therapeutic molecules capable of modulating the NAD biosynthetic pathway in relation to integrin signaling offers new therapeutic perspectives for cardiac pathologies.

## Figures and Tables

**Table 1:** Echocardiographic analysis of control and *Nmrk2 KO* mice at baseline and after 2 weeks of TAC.

Cardiac Parameters	Baseline		TAC Day 15		ANOVA
	Wt	Nmrk2-KO	Wt	Nmrk2-KO	
N	3	5	3	5	
IVSThD (cm)	0.06 ± 0.006	0.06 ± 0.005	0.1 ± 0 ***	0.09 ± 0.008 ***^, #^	i, ¶¶¶
LVEDD (cm)	0.34 ± 0.006	0.34 ± 0.028	0.35 ± 0.015	0.39 ± 0.043	
LVPWThD (cm)	0.07 ± 0.006	0.08 ± 0.008	0.09 ± 0.007	0.08 ± 0.007	¶
IVSThS (cm)	0.11 ± 0.006	0.11 ± 0.007	0.14 ± 0 *	0.12 ± 0.013 ^#^	¶, §
LVESD (cm)	0.18 ± 0.006	0.19 ± 0.024	0.2 ± 0.017	0.27 ± 0.065	
LVPWThS (cm)	0.11 ± 0.010	0.12 ± 0.008	0.14 ± 0.006	0.11 ± 0.010 ^##^	i, §
EDV (mL)	0.1 ± 0	0.11 ± 0.023	0.11 ± 0.0	0.16 ± 0.051	
ESV (mL)	0.02 ± 0	0.02 ± 0.007	0.02 ± 0.007	0.06 ± 0.041	
LVEF (%)	81.7 ± 0.87	81.8 ± 2.47	80.6 ± 2.79	65.8 ± 13.98 *	
FS (%)	44.5 ± 1.12	44.54 ± 2.58	43.3 ± 2.73	32.1 ± 9.54 *^, #^	
SV (mL)	0.08 ± 0	0.09 ± 0.018	0.09 ± 0.006	0.1 ± 0.019	
h/r	0.41 ± 0.021	0.42 ± 0.048	0.55 ± 0.020 *	0.44 ± 0.049 ^##^	¶, §
HR (bpm)	610 ± 12.4	613 ± 15.1	566 ± 12.9	541 ± 55.6	¶
LVMI (mg/g)	2.92 ± 0.320	3.49 ± 0.441	5.01 ± 0.747 ^§§^	5.56 ± 1.059 ^###^	¶¶

Mice series related to Figure 3. Abbreviations: IVSThD and IVSThD, interventricular septum thickness, in diastole and systole, respectively LVEDD and LVESD, left ventricle (LV) end-diastolic and end-systolic diameter; LVPWTh, LV posterior wall thickness; EDV and ESV, end-diastolic and end-systolic volume; LVEF, LV ejection fraction; FS, fractional shortening; h/r, LV mean wall thickness/radius ratio; HR, heart rate in beats per minute. Two-way repeated measure ANOVA for independent samples: i, *p* ≤ 0.05 for the interaction effect; ¶, *p* ≤ 0.05; ¶¶, *p* ≤ 0.01; ¶¶¶ *p* ≤ 0.001 for the TAC effect; § *p* ≤ 0.05, ^§§^ *p* ≤ 0.01 for the genotype effect. Post-hoc Sidak’s multiple comparison test: *, *p* ≤ 0.05; ***, *p* ≤ 0.001 TAC vs baseline within the same genotype; #, *p* ≤ 0.05, ##; *p* ≤ 0.01; ###, *p* ≤ 0.001, Nmrk2-KO mice vs Wt within the TAC group.

## Data Availability

Original data can be provided upon request.

## Supplementary Materials

The following are available online at https://www.mdpi.com/article/10.3390/ijms22073534/s1, Figure S1: title, Table S1: title, Video S1: title.
