# Supplementary material for: NMRK2 Gene Is Upregulated in Dilated Cardiomyopathy and Required for Cardiac Function and NAD Levels during Aging
Source: Int J Mol Sci. 2021 Mar 29;22(7):3534. doi: 10.3390/ijms22073534 (PMC8036583; doi:10.3390/ijms22073534)
Supplement: Supplementary file 1 [file ijms-22-03534-s001.pdf]

The NMRK2 kinase is required to counter eccentric cardiac remodeling and myocardial NAD depletion in aged mice

Cynthia Tannous[1, 2, 3], Robin Deloux[1], Ahmed Karoui[1], Nathalie Mougenot[4], Dean Burkin[5], Jocelyne Blanc[2], Dario Coletti[2], Gareth Lavery[6], Zhenlin Li[2*], Mathias Mericskay[1, *¶]

Supplemental material and methods

X-Gal staining

X-Gal detection of the *LacZ* reporter gene product was performed as follows: The embryo as well as the different organs were fixed 30minutes at 4°C in 1% formaldehyde, 0.02% NP40, 0.01% Sodium deoxycholate in PBS 1X pH 7.4, the washed in PBS 1X solution containing 0.02% NP40, 0.01% and 0.01% Sodium deoxycholate. The coloration was then performed o/n at 30°C with agitation in PBS solution containing 1mg/ml X-Gal, 5mM $MgCl_2$, 5mM $K_3Fe(CN)_6$, $K_4Fe(CN)_6$ 0.02% NP40, 0.01% Sodium deoxycholate[21].

Echocardiography

Transthoracic echocardiography was performed on lightly anesthetized mice given isoflurane (induction with 2% isoflurane 100% O_2 and maintained with 0.5% isoflurane 100% O_2). Non-invasive measurements of left ventricular dimension were evaluated using Doppler echocardiography (Vivid 7 Dimension/Vivid7 PRO; GE Medical Systems) with a probe ultrasound frequency range of 9−14 MHz. The two-dimensionally guided time motion recording mode (parasternal long-axis view) of the left ventricle (LV) provided the following measurements: diastolic and systolic septal (IVS) and posterior wall thicknesses (LVPW); internal end-diastolic (LVEDD) and end-systolic diameters (LVESD); and heart rate. Each set of measurements was obtained from the same cardiac cycle. At least 3 sets of measurements were obtained from 3 different cardiac cycles. LV fractional shortening (FS) was calculated using the formula: (LVEDD–LVESD)/LVEDD×100. LV myocardial volume (LVV), LV end-diastolic volume (EDV), and end-systolic volume (ESV) were calculated using a half-

ellipsoid model of the LV. From these volumes, LV ejection fraction (EF) was calculated using the formula: (EDV-ESV)/EDV×100.

RNA Extraction and Quantification

cDNAs were reverse transcribed from 1.5 µg RNA extracted in TRIZOL (Thermofisher) from cardiac tissue using the superscript II Reverse transcriptase (Life Technologies). Quantitative PCR was carried out on a Light Cycler 480 (Roche Diagnostics) using Fast Start SYBR Green Master (Roche Diagnostics). Quantification of gene expression was calculated as R=2ΔCt (Ref Cp-target Cp), with GAPDH or HPRT used as a reference. Primers were designed using the NCBI Primer Blast Software.

Western Blot Analysis

Proteins were homogenized in a lysis buffer (50 mMTris, 150 mMNaCl, 1% Triton X-100, 0.5% sodium deoxycholate, 0,1% SDS, 1 mM EDTA, pH 7.4) containing protease inhibitors (1mM Phenylmethylsulfonyfluoride, 5mM EDTA, E64, Aprotinin) and phosphatases inhibitors (1mM Sodium Orthovanadate, 40 mM β-Gycerophosphate, 30mM NaF, 20 mM Sodium pyrophosphate). Equal amounts of proteins (20 to 30 µg) were separated on one-dimensional PAGE using the NuPAGE® Novex® 4–12% Bis-Tris Bolt Gel from Invitrogen according to the manufacturer's instructions. Proteins were transferred onto nitrocellulose membranes (Hybond C, Amersham Biosciences) and the blots were saturated in TBS buffer (20 mMTris, pH 7.5, 136.8 mMNaCl, and 0.1% (v/v) Tween 20) containing 5% (w/v) milk. Proteins were detected by overnight incubation at 4°C with primary antibodies, followed by HRP-labeled secondary antibodies (1:2500) and then exposed to ECL (OzymeRevelBlot® Plus) and scanned on FUJIFILM LAS3000 systems.

Target Protein	Antibody Supplier. ref #	Dilution
NMRK2	Gareth Lavery's lab ([1]	1:500
Extracellular signal-regulated kinase (ERK)	Cell signaling #4695	1:1000
Phospho-ERK	Cell signaling #4370S	1:1000

Paxillin (PAX)	Cell signaling #2542S	1:1000
Phospho-PAX	Cell signaling #2541S	1:1000
Integrin α7	Dean Burkin's lab	1:200
GAPDH	Sigma #G9545	1:3000

Immunofluorescence staining

Hearts were harvested after cervical dislocation. Frozen heart sections (8µm) were fixed in 3.7% formaldehyde in PBS at room temperature (RT) for 10 minutes followed by permeabilization in PBS/Triton X100 (0.2%) for 10 minutes. Saturation of non-specific sites was obtained with 2% BSA and 10% of goat serum. Sections were incubated o/n at 4°C with primary antibodies diluted in saturation solution. The day after, sections were washed and incubated with Cy3-coupled secondary antibodies (1:400, Jackson ImmunoResearch) or Alexa 488 coupled secondary antibodies (Invitrogen, 1:400) and Cy3 coupled anti rat (Jackson ImmunoResearch, 1:500) or Cy3 coupled anti rabbit (Confocal images were acquired on LEICA with identical gain and offset parameters for all samples.

Target Protein	Antibody Supplier. ref #	Dilution
NMRK2 (MIBP)	MBL International Corp. #K0099-3, mouse monoclonal 5B4.7 (immunofluorescence)	1:100
Vinculin (FITC coupled anti-hVIN-1)	Sigma mouse monoclonal, F7053	1:500
Integrin α7	Dean Burkin's lab, rabbit polyclonal	1:500
Integrin β1	Sigma-Aldrich, Millipore MAB1997, clone MB1.2	1:250
Laminin α2	Sigma rat monoclonal, clone 4H8-2, #L0663	1:200

NAD extraction and quantification

NAD levels were measured using a colorimetric approach. Weighted frozen heart tissue (20 to 30 mg) were crushed with silica beads in a Precellys homogenizer by 2 cycles of 8 seconds (Bertin instruments, Montigny-le-Bretonneux FRANCE) in 10 volumes (µl/mg) of buffered ethanol (BE) (75% Ethanol, 25% HEPES 10 mM pH7.1). Extracts were heated for 3 minutes at 80°C, crushed a second time, briefly cold on ice and centrifuged 20 minutes at 16000 g. For each sample, 25 µL of extracts were quantified in duplicates by the addition of 100µL of reaction buffer (600 mM ethanol, 0.5 mM 3-(4.5-dimethyltiazol-2-yl)-2.5-diphenyltetrazolium bromide (MTT), 2 mM phenazineethosulfate (PES), 120 mM Bicine (pH 7.8), yeast alcohol dehydrogenase (SIGMA A3263>300 u/mg) 0.05 mg/ml). Kinetics of the reaction (OD at 570 nm, every 30 seconds for 40 minutes) was followed on a TECAN INFINITE F500 microplate reader. NAD concentration were quantified by comparison to a range of standard NAD^+ concentration (linear regression curve equation method). In this recycling assay, both NAD^+ and NADH are indistinctly detected and it is the complete pool of extracted NAD that is measured. Data are expressed in pmole of NAD / mg of tissue.

Transmission Electron Microscopy (TEM)

For transmission electron microscopy, the cells were washed in 0.1 M phosphate buffer, pH 7.4 and fixed in 2% glutaraldehyde for 1 h at room temperature. The samples were first washed in 0.1 M phosphate buffer and then in bidistilled water and finally post-fixed in 1% osmium-bidistilled water for 1 h at room temperature. After washes in bidistilled water, the samples were dehydrated in increasing concentrations of ethanol, infiltrated in 1:1 ethanol:epon resin for 1 h and finally 100% epon resin for 48 h at 60 °C for polymerization. Seventy-nm-thick sections were cut with an ultracut UCT microtome (LEICA) and picked up on copper rhodium-coated grids. Grids were stained for 2 min in Uranyless (DELTA Microscopies) for 5 min in 0.2% lead citrate. Grids were analyzed on an electron microscope (EM 912 OMEGA, ZEISS) at 80 kV, and images were captured with digital camera (Side-Mounted TEM CCD, Veleta 2kx2k). The software used is iTEM.

Adult cardiomyocytes isolation and morphometric analayses

Mice were anesthetized by pentobarbital (150 µg/g of body weight). Adult heart were cannulated at the ascending aorta and perfused for 8 to 10 minutes (2.5 ml/min) in a buffer solution (Na Cl 113 mM, KCk 4.7 mM, MgSO4 1.2 mM, KH2PO4 0.6 mM, NaH2PO4 0.6 mM, HEPES 10 mM, NaHCO3 1.6 mM, Taurine 30 mM, Glucose 20 mM) containing Liberase TM Research Grade (Roche ref : 054001127001) at 1:60 dilution. Ca^{2+} was progressively reintroduced in medium up to 1 mM. Cardiomyocytes were plated on laminin coated plates and fixe in 4% paraformaldehyde before image

acquisition. Cardiomyocytes contours were delineated manually on Image J and value for area, Feret length and minFeret width were obtained.

Supplemental references

1. Fletcher, R.S.; Ratajczak, J.; Doig, C.L.; Oakey, L.A.; Callingham, R.; Da Silva Xavier, G.; Garten, A.; Elhassan, Y.S.; Redpath, P.; Migaud, M.E.; et al. Nicotinamide riboside kinases display redundancy in mediating nicotinamide mononucleotide and nicotinamide riboside metabolism in skeletal muscle cells. *Mol Metab* 2017, *6*, 819–832, doi:10.1016/j.molmet.2017.05.011.

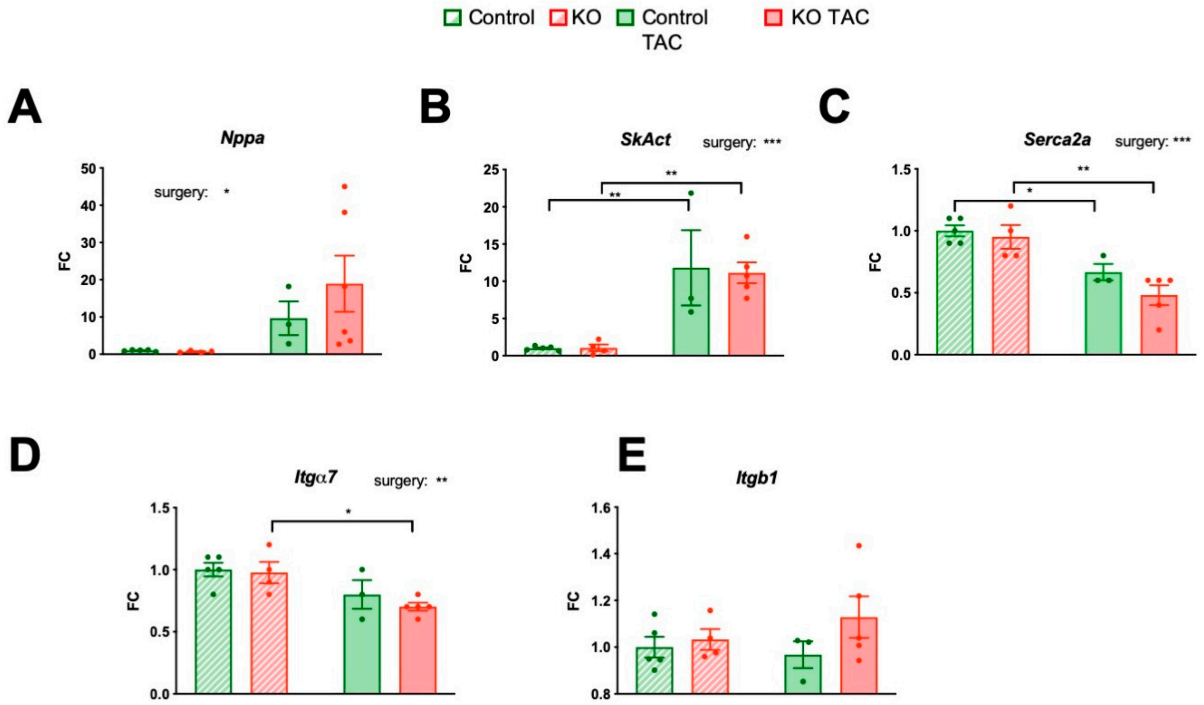

Supplementary Figure 1. Regulation of cardiac genes in controls and Nmrk2-KO mice at baseline and after TAC. Relative gene expression by RT qPCR. **(A)** natriuretic peptide precursor A. **(B)** α skeletal actin. **(C)** Sarco/endoplasmic reticulum Ca^{2+} pump ATPase cardiac muscle, slow twitch isoform 2a. **(D)** Integrin α7. **(E)** Integrin β1. Individual data are plotted as dots. Data are normalized on *Hprt* reference mRNA level and expressed as fold change over mean control value +/- SEM. Two-way ANOVA for independent samples: *, p≤ 0.05; **, p≤ 0.01; ***, p≤ 0.001; ****, p≤ 0.0001, for indicated factor as indicated next to graph title. Tukey's multiple comparison test: *, p≤ 0.05; **, p≤ 0.01; TAC vs baseline within the same genotype. This figure accompanies **Figure 3**.

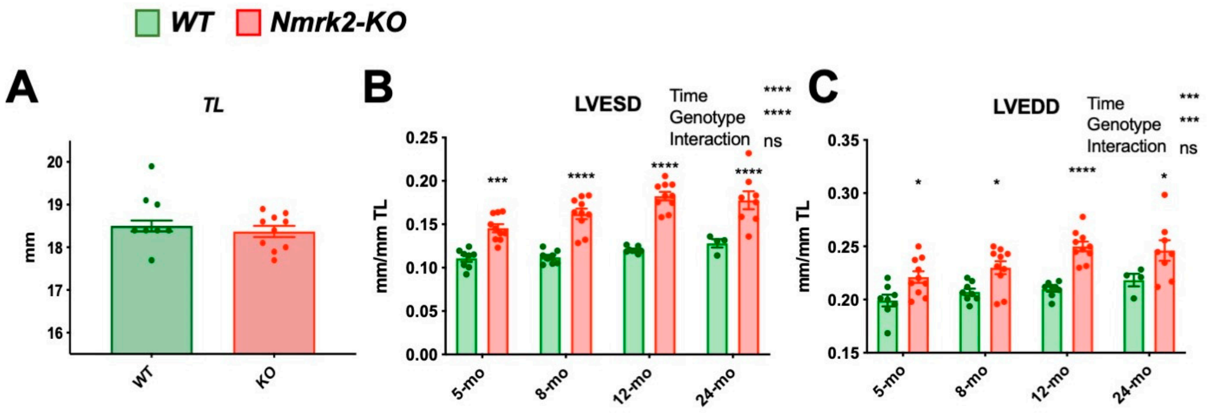

Supplementary Figure 2. LV echocardiography normalized on tibia length in the aging series. **(A)** Tibia length was measured post-mortem**.** **(B)** LV end-systolic diameter and **(C)** LV end-diastolic diameter normalized on tibial length. Individual data are shown by dots. Bars and error bars represent the mean value ± SEM. Statistics: Mixed model analysis by restricted maximum likelihood (REML). Fixed factors p-value are indicated next to the graph title: *, p≤ 0.05; **, p≤ 0.01; ***, p≤ 0.001; ****, p≤ 0.0001 for time and genotype. Sidak's multiple comparisons test p-value: *, p≤ 0.05; ***, p≤ 0.001; ****, p≤ 0.0001, between control and *Nmrk2-KO* mice within each age group. This figure accompanies **Figure 4**.

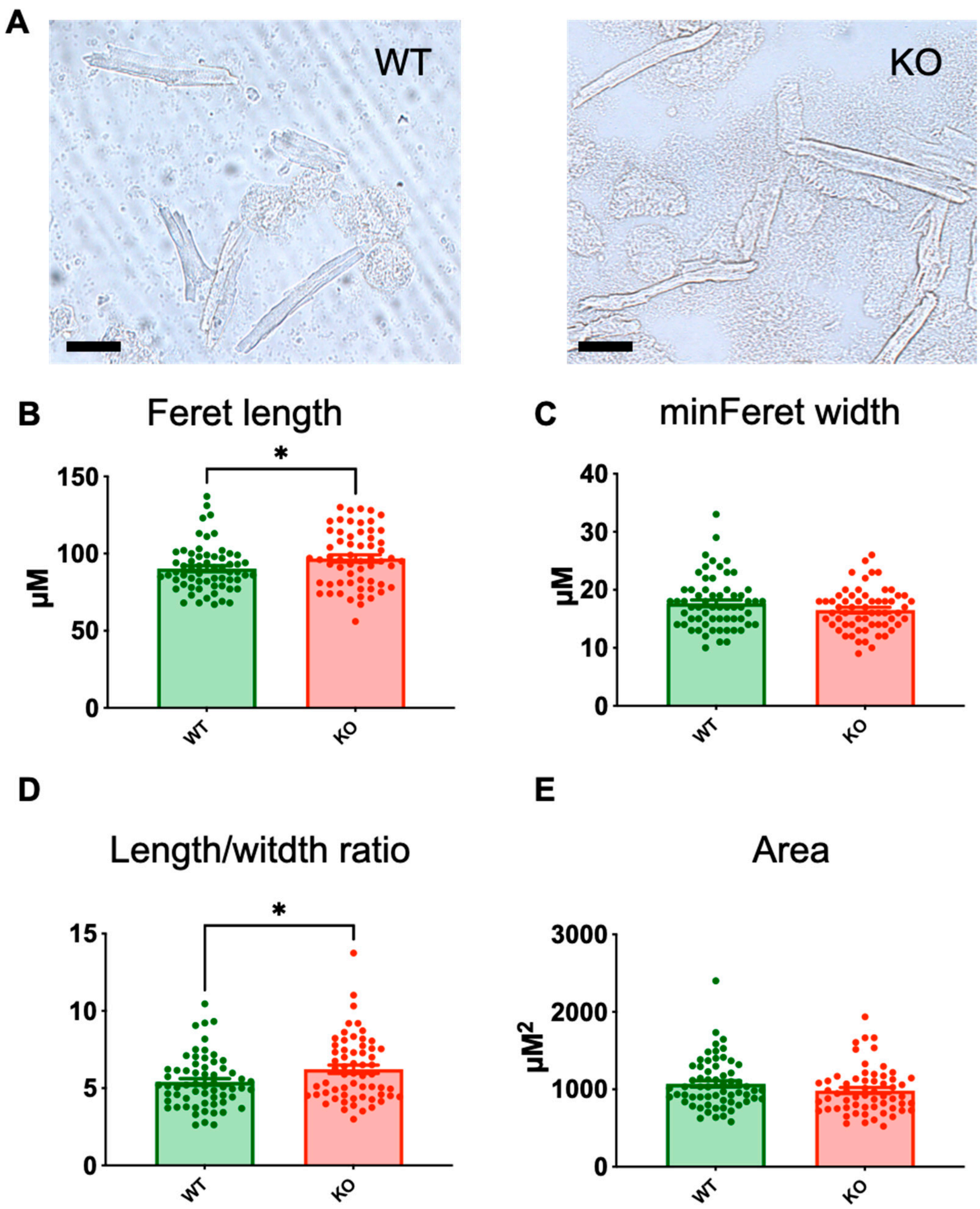

Supplementary Figure 3. Morphometric analysis of isolated adult cardiomyocytes. Adult cardiomyocytes were isolated from 4-month-old *Nmrk2-KO* and wildtype mice and measured using Image J software. (**A**) Representative picture of cardiomyocytes after isolation. Scale bars= 30 µm.(**B**) Feret length. (**C**) minFeret. (**D**) Length/width ratio. (**E**) Area. Technical replicates (n>60) were obtained from n=3 mice/group. Data are expressed as the mean +/- SEM. T-test. *, p<0.05.

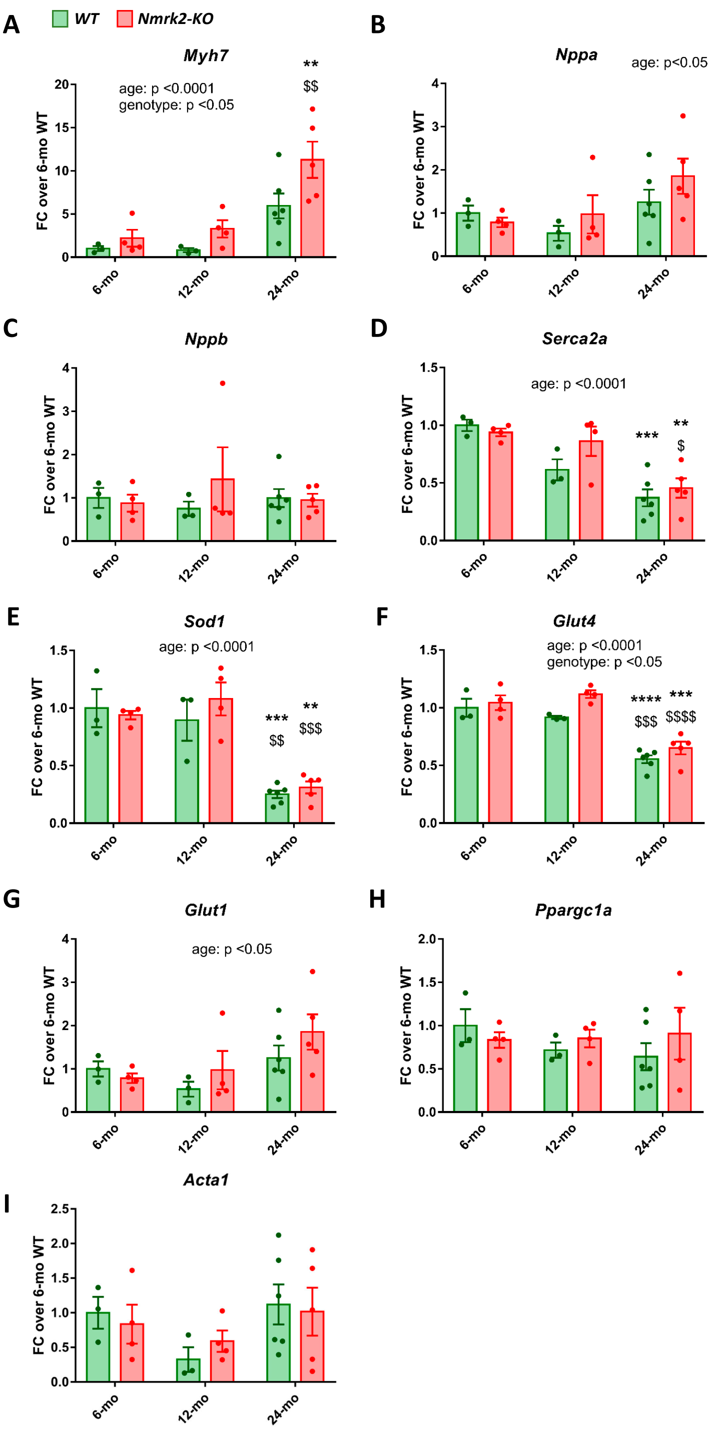

Supplementary Figure 4. Time course analysis of cardiac genes expression during aging. Cardiac RNA was extracted at the age of 6, 12 and 24 months in control and mutant mice and analyzed by RT-qPCR. Data are normalized on *Gapdh* reference mRNA level. Genes involved in cardiac stress and heart failure.: **(A)** *βeta Myosin Heavy Chain (Myh7).* **(B)** *Natriuretic peptide precursor A.* **(C)** *Natriuretic peptide precursor B.* **(D)** *Sarco/endoplasmic reticulum*

Ca^{2+} pump ATPase cardiac muscle, slow twitch isoform 2a. **(E)** *Superoxide dismutase 1*. **(F)** *Glucose transporter 4*. **(G)** *Glucose transporter 1*. **(H)** *PPARγ coactivator 1α*. **(I)** *Skeletal α actin (Acta1).* Individual data are shown by dots. Bars and error bars represent the fold change over mean control value at 6 months ±. Statistics: Two-way ANOVA for independent samples: Factors p values are indicated are indicated next to the graph title. On bars, Post-hoc Tukey's multiple comparison test: **, p≤ 0.01; ***, p≤ 0.001; ****, p≤ 0.0001, 24 months vs 6 months within the same genotype; $, p≤ 0.05, $$; p≤ 0.01; $$$; p≤ 0.001; $$$$; p≤ 0.0001, 24 months vs 12 months within the same genotype.

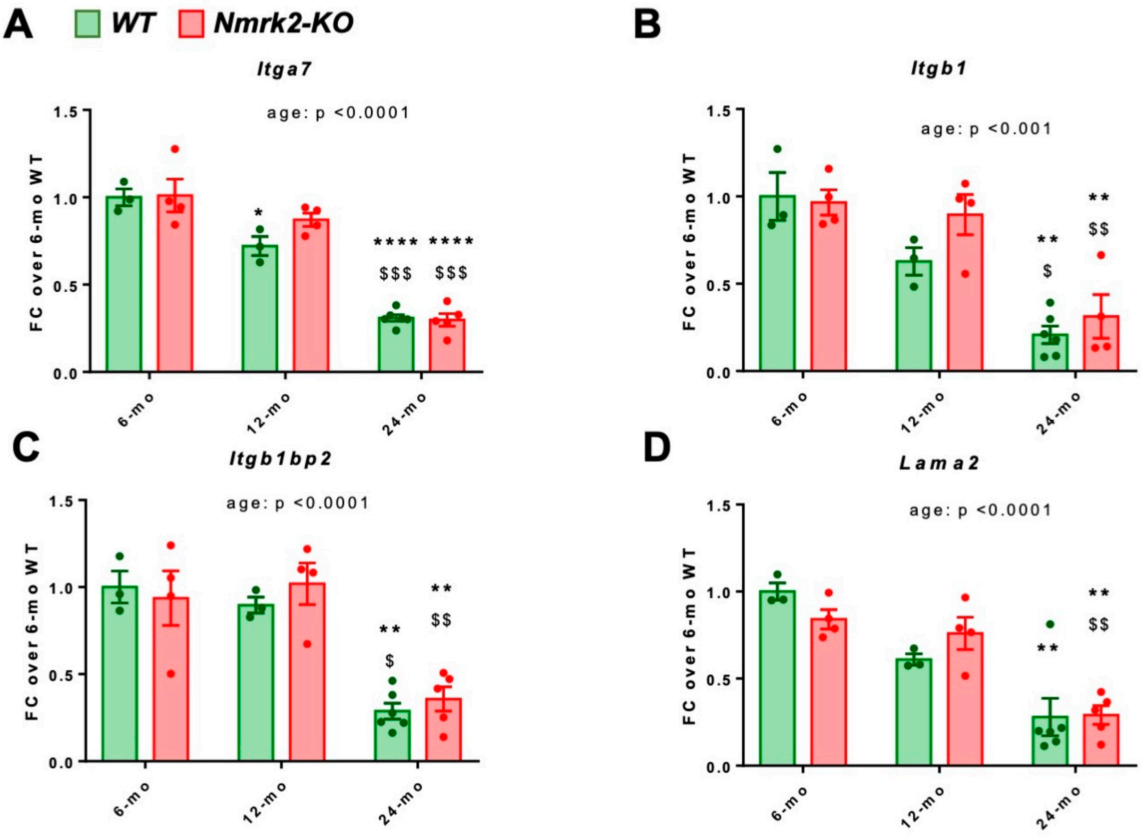

Supplementary Figure 5. Time course analysis of integrin-related genes expression during aging. Cardiac RNA was extracted at the age of 6, 12 and 24 months in control and mutant mice and analyzed by RT-qPCR. Data are normalized on *Gapdh* reference mRNA level. **(A)** *Integrin $\alpha 7$.* **(B)** *Integrin $\beta 1$.* **(C)** *Integrin $\beta 1$ binding protein 2 (Melusin)*. **(D)** *Laminin $\alpha 2a$*. Individual data are shown by dots. Bars and error bars represent the fold change over mean control value at 6 months ± SEM. Statistics: Two-way ANOVA for independent samples: Factors p values are indicated are indicated next to the graph title. On bars, Post-hoc Tukey's multiple comparison test: **, p≤ 0.01; ***, p≤ 0.001; ****, p≤ 0.0001, 24 months vs 6 months within the same genotype; $, p≤ 0.05, $$; p≤ 0.01; $$$; p≤ 0.001; $$$$; p≤ 0.0001, 24 months vs 12 months within the same genotype.

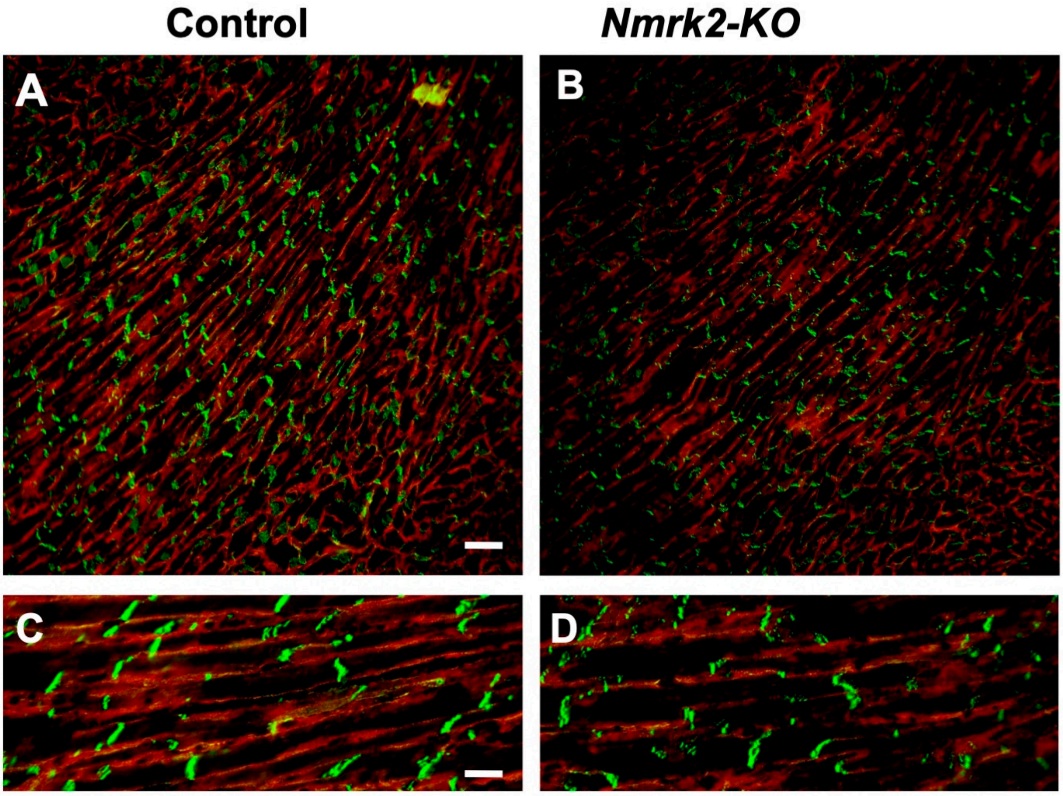

Supplementary Figure 6. Integrin β1 expresssion pattern at 24 months. Frozen heart cross sections of 24 month-old control and *Nmrk2-KO* mice were stained with anti-integrin β1 antibody (red staining) and vinculin for intercalated disks (in green). (**A-B**) Low magnification in the free wall of the left ventricle. Scale bar = 50 µM. (**C-D**) High magnification. Scale bar = 10 µM. Pictures are representative of n=3 mice/group.
